# Effect of *TRPM8* and *TRPA1* Polymorphisms on COPD Predisposition and Lung Function in COPD Patients

**DOI:** 10.3390/jpm11020108

**Published:** 2021-02-08

**Authors:** Denis E. Naumov, Olesya O. Kotova, Dina A. Gassan, Ivana Y. Sugaylo, Evgeniya Y. Afanas’eva, Elizaveta G. Sheludko, Juliy M. Perelman

**Affiliations:** 1 Far Eastern Scientific Center of Physiology and Pathology of Respiration, Laboratory of Molecular and Translational Research, 675000 Blagoveshchensk, Russia; foxy_voxy_on@mail.ru (O.O.K.); dani-shi@mail.ru (D.A.G.); ivanka_888@mail.ru (I.Y.S.); evgeniyananev@yandex.ru (E.Y.A.); liza.sheludko@mail.ru (E.G.S.); 2 Far Eastern Scientific Center of Physiology and Pathology of Respiration, Laboratory of Functional Research of the Respiratory System, 675000 Blagoveshchensk, Russia; jperelman@mail.ru

**Keywords:** COPD, lung function, smoking, TRPM8, TRPA1, polymorphism

## Abstract

Certain transient receptor potential (TRP) channels including *TRPM8* and *TRPA1* are widely expressed in the respiratory tract and have been shown to be the receptors of cigarette smoke and particulate matter—the main causative factors of chronic obstructive pulmonary disease (COPD). The aim of the study was to investigate the effect of *TRPM8* and *TRPA1* polymorphisms on COPD predisposition and lung function in COPD patients. The study enrolled 143 COPD patients and 104 smokers with post-bronchodilator forced expiratory volume in one second (FEV1)/forced vital capacity (FVC) > 70%. Lung function was measured by spirometry. *TRPM8* and *TRPA1* polymorphisms were genotyped by LATE-PCR. None of the polymorphisms significantly influenced COPD predisposition after correction for covariates and multiple testing. Among COPD patients, the TT genotype of *TRPA1* rs7819749 was significantly associated with higher degree of bronchial obstruction. In addition, we established that carriers of the C allele of *TRPM8* rs11562975 more commonly had post-bronchodilator FEV1 < 60% (OR 3.2, 95%CI (1.14–8.94), *p* = 0.03) and revealed the effect of *TRPA1* rs959976 and *TRPM8* rs17865682 on bronchodilator response in COPD. Thus, the obtained results suggest possible involvement of *TRPM8* and *TRPA1* in COPD pathogenesis, indicating the necessity to further investigate their functional role in this pathology.

## 1. Introduction

According to the Global Initiative for Chronic Obstructive Lung Disease (GOLD) guidelines, chronic obstructive pulmonary disease (COPD) is a common, preventable and treatable disease characterized by persistent respiratory symptoms and airflow limitation due to airway and/or alveolar abnormalities usually caused by significant exposure to noxious particles or gases [1]. In 2010, the estimated worldwide COPD prevalence was 11.7% (8.4%–15.0%), with the highest values in the USA (15.2% (14.9%–15.5%)) and the lowest in South-East Asia (9.7% (9.3%–10.1%)) [2]. An increase of 68.9% in COPD cases has been observed over a twenty-year period worldwide, mainly at the expense of urban populations [2]. Although the disease is defined as ‘treatable’, the effectiveness of the modern therapeutic approaches remains relatively limited. Despite proper treatment, some patients continue to experience frequent exacerbations with progressive loss of lung function which eventually leads to disability and premature mortality. Since 2010, COPD has become the third leading cause of death, with mortality rate up to 141 per 100,000 person-years, in some countries [3]. The presence of different clinical phenotypes of COPD with corresponding underlying pathogenesis implies the urge for a personalized approach in disease management and the discovery of novel pharmacological targets [4].

Tobacco smoking is widely considered to be among the major risk factors for COPD. However, in ever-smokers, only 17.8% were reported to have COPD. Nevertheless, among non-smokers the prevalence of COPD is significantly lower (6.4%) [5]. In the latter category of patient, a substantial role in COPD development belongs to occupational exposures and general air pollution, including traffic, biomass and secondhand smoke [6].

Usually, cigarette smoke and air pollutants are thought to exert their effect on the airways in a non-specific manner, promoting reactive oxygen species (ROS) production, release of damage-associated molecular pattern molecules and activation of inflammatory cells. However, a growing body of data generated during the latest decade has provided us with new ideas of how transient receptor potential (TRP) channels may mediate the effect of cigarette smoke and particulate matter on living cells and so be potentially important in COPD pathogenesis.

TRP channels represent a family of receptor proteins sensitive to a variety of exo- and endogenous stimuli of a physical and chemical nature [7]. Up to date, four main TRP channels have been established as receptors of smoke constituents and particles: TRPM8, TRPA1, TRPV1 and TRPV4. These channels may be found throughout the respiratory system on different cell types, including nerve endings, bronchial and alveolar epithelium, macrophages and other cells of the immune system [8]. Among them, *TRPA1* is the most consistently reported receptor of cigarette smoke, sensitive to such toxic carbonyl compounds as formaldehyde and acrolein [9]. Along with TRPA1, TRPM8, TRPV1 and TRPV4 were also found to be sensitive to cigarette smoke and particulate matter [10,11,12,13,14]. Besides, TRPV1, TRPM8 and, to a lesser extent, *TRPA1* were capable of activation under exposition with coal fly ash [15].

There are few studies which have previously investigated an association between TRP genetic polymorphisms and COPD. The first, conducted by Zhu et al., utilized both family-based and case-control design and revealed four single-nucleotide polymorphisms (SNPs) of *TRPV4* persistently associated with COPD [16]. Another two studies established that the polymorphisms of *TRPM8* and *TRPV1* may influence COPD predisposition in the Chinese Han population [17,18]. However, *TRPM8* or *TRPV1* variants that showed the most significant associations are almost absent in Europeans due to extremely low minor allele frequency, so it is difficult to replicate or extrapolate the obtained results. Considering these facts, the aim of the present study was to investigate the effect of *TRPM8* and *TRPA1* polymorphisms on COPD predisposition and lung function in a European population.

## 2. Materials and Methods

### 2.1. Study Population

A total of 247 unrelated subjects including 143 COPD patients from the inpatient department of the Far Eastern Scientific Center of Physiology and Pathology of Respiration and 104 healthy controls were enrolled in the study. All the enrolled persons were Europeans living in the Amur Oblast of Russia. COPD was diagnosed by standard criteria (symptoms, risk factors, post-bronchodilator forced expiratory volume in one second (FEV1)/forced vital capacity (FVC) < 70%) according to GOLD guidelines [1]. Subjects for the control group were recruited from active smokers with FEV1/FVC > 70% undergoing regular medical examination in the outpatient department. Subjects without smoking history or those who had any other respiratory pathology, besides COPD, were not included in the study. The study was approved by the local Ethics Committee of the Far Eastern Scientific Center of Physiology and Pathology of Respiration (Ethics approval No. 131; 11 January 2019) and conducted in accordance with the Declaration of Helsinki—Ethical Principles for Medical Research Involving Human Subjects (revised in 2013). An informed consent was obtained from all the participants before inclusion in the study.

### 2.2. Lung Function Measurement

Lung function was measured by standard spirometry using an Easy-on PC spirometer (ndd Medizintechnik AG, Zurich, Switzerland) in accordance with American Thoracic Society/European Respiratory Society (ATS/ERS) guidelines [19]. Values of forced expiratory volume in one second (FEV1), forced vital capacity (FVC), FEV1/FVC ratio, peak expiratory flow (PEF), forced expiratory flow at 50% and 75% of FVC (FEF50 and FEF75) and maximum mid-expiratory flow (MMEF) were determined before and 15 min after four puffs of salbutamol (400 µg). Positive response to bronchodilator was defined as an increase in FEV1 by ≥12% or ≥200 mL 15 min after salbutamol inhalation.

### 2.3. Genotyping

**Figure 1:**
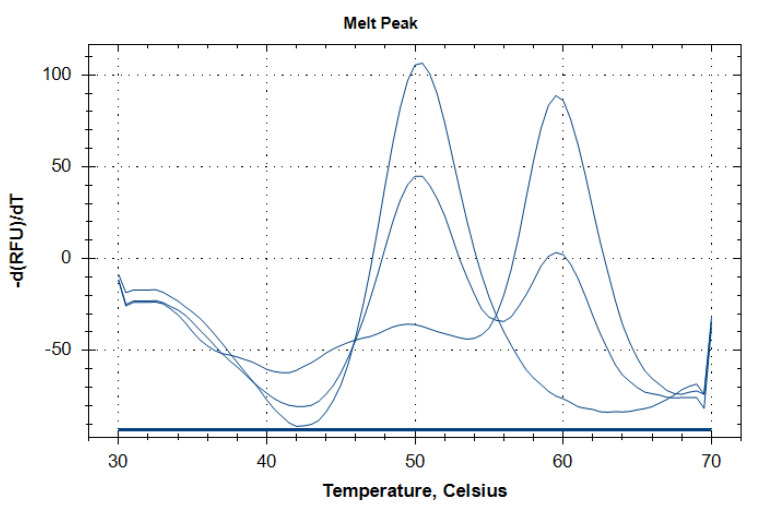
Representative melting curves corresponding to three different genotypes of *TRPM8* rs2052030 polymorphism. Single peaks at 60 °C and 50 °C correspond to the CC and GG genotypes, respectively. Simultaneous presence of both peaks indicates the heterozygous CG genotype.

Genomic DNA was extracted from whole blood using the DNK-Extran-1 kits (Syntol LLC, Moscow, Russia). Five *TRPM8* and six *TRPA1* polymorphisms were selected based on the combined approach considering minor allele frequency (>0.05), evidence of associations from the previous studies and functional significance predicted by the bioinformatic tool SNP Function Prediction [20]. Brief characteristics of the selected polymorphisms are summarized in Table 1. The polymorphisms were genotyped by LATE-PCR with melting analysis of molecular beacon probes [21]. PCR was performed on CFX96 Touch Real-Time PCR System (Bio-Rad, Hercules, CA, USA) using PCR-RV reagent kits (Syntol LLC, Moscow, Russia). The reaction mixture contained: DNA—50 ng, 1X PCR-buffer, MgCl_2_—2.5 mM, dNTP—0.25 mM, limiting primer—0.02 µM, excessive primer—0.5 µM, molecular beacon probe—0.5 µM, Hot-Start Taq-polymerase—1 U, ddH_2_0—up to 25 µL. Thermal cycling was performed under the following conditions for each polymorphism: initial denaturation at 96 °C for 1.5 min; first 25 cycles—denaturation at 96 °C for 1 s, annealing/extension at primer-specific temperature (Ta_1_) for 15 s; following 45 cycles—denaturation at 96 °C for 1 s, annealing/extension at primer-specific temperature (Ta_2_) for 15 s; final extension at 72 °C for 1 min. Ramp rate was set to 3 °C/s. Melting analysis of molecular beacon was performed immediately after the thermal cycling but was preceded by a denaturation step at 96 °C for 3 min and hybridization at 30 °C for 3 min. Then, PCR tubes were heated stepwise from 30 °C to 70 °C at a rate of 0.5 °C per step with 5 s hold at each temperature. A fluorescent signal in the FAM channel was detected at the end of each step. The form of generated melting curves allowed for allelic discrimination relying on the corresponding peaks on the negative first derivative of the fluorescence-versus-temperature plots (Figure 1). Detailed information on polymorphism genotyping including oligonucleotide sequences, concentrations and thermal cycling conditions is available in Supplementary Table S1.

### 2.4. Statistical Analysis

Statistical analysis was performed using Statistica version 10.0 software (StatSoft Inc., Tulsa, OK, USA). Kolmogorov–Smirnov and Shapiro–Wilk tests were used to determine whether the data are normally distributed. Due to non-normal distribution, quantitative data are presented as median (interquartile range). Mann-Whitney U test was used for pair-wise comparisons of quantitative variables. Frequency distribution of the genotypes and alleles was presented as percentage. Hardy–Weinberg equilibrium between observed and expected genotype frequencies was evaluated by Pearson’s chi-square test. Associations between categorical variables were assessed by Pearson’s chi-square test or Fisher’s exact test. Adjustment for covariates was performed in logistic regression analysis. Benjamini-Hochberg False Discovery Rate (FDR) was used for multiple testing correction. *p*-value < 0.05 was considered statistically significant and FDR corrected *p*-value < 0.25 was considered as a trend.

## 3. Results

### 3.1. Clinical and Functional Characteristics of the Studied Subjects

Differences in clinical parameters, smoking history and lung function between the case and control groups are shown in Table 2. COPD patients were significantly older, had longer exposition to cigarette smoke, marked bronchial obstruction and higher response to bronchodilator.

### 3.2. Role of TRPM8 and TRPA1 Polymorphisms in COPD Predisposition

Genotype frequencies for each of the studied polymorphisms were in Hardy–Weinberg equilibrium both in case and control groups. Generally, the differences in genotype frequencies for the studied polymorphisms were tested in the codominant genetic model. However, in cases in which less than five minor allele homozygotes were detected in either group, they were merged with heterozygotes and the dominant genetic model was employed (Table 3).

We found that prior to correction for multiple comparisons or possible confounders *TRPM8* rs2052030 was significantly associated with COPD. Despite the fact that the significance of this association was lost after the adjustments, we additionally estimated the potential effect of rs2052030 on COPD predisposition under other genetic models (Table 4).

As a result, it was established that the effect of *TRPM8* rs2052030 on COPD was best described by the over-dominant model. The heterozygous CG genotype was prevalent among control group but relatively underrepresented in the studied COPD patients. This association retained its significance after adjustment for sex, age and pack-year index, but was not significant after FDR correction for multiple comparisons (*p* = 0.22).

### 3.3. Effect of TRPM8 and TRPA1 Polymorphisms on Lung Function in COPD

**Figure 2:**
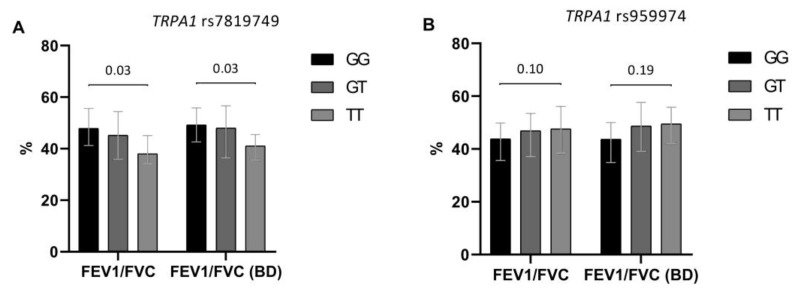
Effect of *TRPA1* rs7819749 (**A**) and *TRPA1* rs959974 (**B**) polymorphisms on baseline and post-bronchodilator (BD) FEV1/FVC in the studied COPD patients (*n* = 143). The results are presented as median, lower and upper quartiles. Denoted levels of significance are FDR corrected and correspond to the differences in FEV1/FVC between homozygous genotypes.

We also tested *TRPM8* and *TRPA1* polymorphisms for their potential effect on baseline and post-bronchodilator lung function. *TRPA1* rs7819749 and *TRPA1* rs959974 appeared to have the most marked influence on the degree of bronchial obstruction. The T allele of *TRPA1* rs7819749 had detrimental effect on lung function in COPD. Generally, the heterozygotes and, especially, TT homozygotes had lower values of spirometric parameters than the patients with the GG genotype. Similar effects were observed for *TRPA1* rs959974 polymorphism with probable pathogenic effect of the G allele. However, in this case there was only a trend for association (Figure 2).

**Figure 3:**
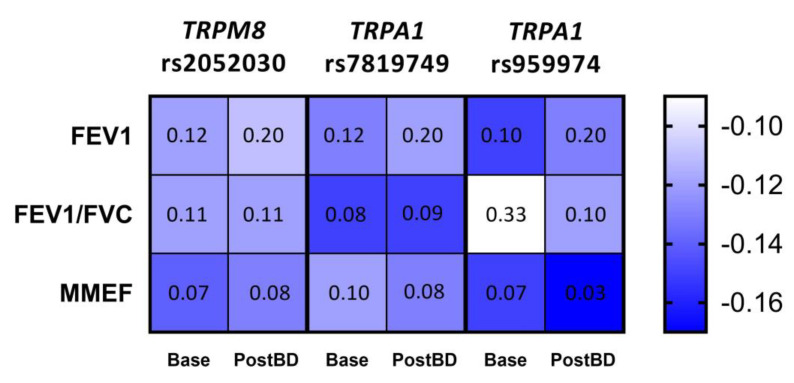
Correlation between the number of risk alleles for *TRPM8* rs2052030, *TRPA1* rs7819749 and *TRPA1* rs959974 polymorphisms and spirometric parameters of the studied COPD patients measured at baseline (Base) and after the inhalation of bronchodilator (PostBD). The values of Kendall’s τ coefficient are color-coded; each cell contains the corresponding FDR *p*-value.

Given the apparent trend of the relationship between lung function parameters and the number of risk alleles for each of the three polymorphisms, we applied Kendall’s tau test to measure the significance of these correlations. As a result, we established a tendency to enhanced bronchial obstruction with the increase in the number of risk alleles for *TRPM8* rs2052030 (G allele), *TRPA1* rs7819749 (T allele) and *TRPA1* rs959974 (G allele). The obtained correlations and FDR corrected *p*-values are shown in Figure 3.

Despite that there were no obvious and strong associations with lung function for *TRPM8* rs11562975 in COPD, we took a closer look at this polymorphism since its influence on FEV1 and FEV1/FVC was established in our previous studies in asthma patients, including those with smoking history > 10 pack-years [28,33]. In accordance with these data, we noticed that in COPD patients the C allele of *TRPM8* rs11562975 had a trend to association with lower post-bronchodilator FEV1/FVC (GG—48.9 (41.4–55.8)%, GC—45.7 (38.1–50.9)%, CC—35.2 (32.7–37.7)%). The CC genotype seemed to have the most profound effect on FEV1/FVC; however, this was not significant due to the low number of observations (*n* = 2). At the same time, analysis carried out in the dominant genetic model (GG vs. GC+CC) revealed the borderline significance for between-genotype difference of FEV1/FVC (48.9 (41.4–55.8)% vs. 43.9 (37.3–49.8)%, *p* = 0.05). Next, we tested the association of *TRPM8* rs11562975 with post-bronchodilator FEV1 < 60%. This cut-off value was set to correspond with the previous finding in smoking asthma patients. The GC and CC genotypes in combination were found in 26.6% of the patients with FEV1 < 60% and only in 10.2% of the patients with FEV1 ≥ 60% and so increased the odds of having FEV1 < 60% (OR 3.2, 95%CI (1.14–8.94), *p* = 0.03). This association remained significant after correction for sex, age and pack year index (adjusted OR 3.3, 95%CI (1.15–9.67), *p* = 0.03). Unlike in COPD patients, we did not find any associations of the studied polymorphisms with lung function parameters in healthy smokers.

### 3.4. Associations of TRPM8 and TRPA1 Polymorphisms with the Airway Response to Bronchodilator

In the COPD group, 58.7% of patients had positive response to salbutamol, which is considered to be associated with better prognosis in terms of reduced risk of severe acute exacerbations [34]. The *TRPA1* rs959976 polymorphism significantly influenced the response both qualitatively and quantitatively. The heterozygous AG genotype prevailed among the patients with positive response (40.5% vs. 17.0%) while the AA and GG genotypes were more frequent in in non-responders (OR 3.3, 95%CI (1.49–7.69), *p* = 0.003; FDR *p* = 0.02). This effect was independent of sex, age, pack-year index and baseline FEV1 (adjusted OR 3.6, 95%CI (1.51–8.33), *p* = 0.003). Carriers of the heterozygous genotype had the highest bronchodilator response (ΔFEV1 17.3 (12.1–27.9)%) when compared with the AA (12.1 (4.2–22.8)%, *p* = 0.02) or GG genotypes (2.7 (0.1–23.0)%, *p* = 0.04), whereas the difference between the homozygous genotypes was not significant.

Polymorphism rs17865682 of *TRPM8* gene also affected salbutamol response, measured qualitatively. The AA genotype more frequently occurred in the group with positive response (83.3% vs. 59.3%) while the AG and GG genotypes were prevalent among non-responders (33.9% vs. 15.5%, 6.8% vs. 1.2%, respectively). This association was significant after analysis under the dominant model (AA vs. AG+GG). Carriage of the AG or GG genotype increased the odds of being unresponsive to salbutamol in the COPD patients (OR 3.4, 95%CI (1.59–7.69), *p* = 0.002; FDR *p* = 0.02), even after correction for sex, age, pack-year index and baseline FEV1 (adjusted OR 4.2, 95%CI (1.79–10.0), *p* < 0.001). Nevertheless, the difference in ΔFEV1 between the AA and AG+GG carriers showed only a trend toward statistical significance (15.7 (6.0–23.8)% vs. 8.8 (2.7–17.6)%, *p* = 0.06).

## 4. Discussion

Considering the strong evidence for the role of smoking and particulate matter in COPD pathogenesis, the study of molecular mechanisms related to the reception of these detrimental environmental factors is highly justified. TRPM8 and TRPA1 cation channels are polymodal receptors widely expressed in the respiratory tract and sensitive to cigarette smoke and particulate matter. Indeed, according to the previously conducted research, TRPM8 and TRPA1 are likely to be involved in the development of chronic obstructive respiratory pathology. Expression of TRPM8 was repeatedly reported to be upregulated in the respiratory tract of asthma and COPD patients both at mRNA and protein level [35,36,37]. Besides smoke, this channel is also capable of activation by cold temperature and menthol [7]. Stimulation of TRPM8 in the airways is typically accompanied by inflammatory reaction including cytokine production, cellular infiltration and mucus hypersecretion [35,37,38,39]. As compared to TRPM8, the TRPA1 channel, aptly named ‘a gatekeeper of inflammation’ [40], is less studied in real clinical respiratory pathology, probably due to its limited expression in the airway epithelium. At the same time, TRPA1 is abundantly expressed in nerve endings, alveolar epithelium and fibroblasts where it mediates neurogenic and non-neurogenic inflammation upon activation [41,42].

In the present study we investigated the role of *TRPM8* and *TRPA1* genetic polymorphisms in the development of COPD in smoking subjects and explored their effect on lung function in COPD patients. We established that *TRPM8* rs2052030 polymorphism may influence predisposition to COPD development in the over-dominant genetic model. Carriers of the heterozygous CG genotype were relatively protected against COPD in spite of smoking. This effect was independent of sex, age or smoking history, but was not significant after correction for multiple testing. Previously, the only relevant study conducted by Xiong et al. [17] in the Chinese Han population also revealed several *TRPM8* polymorphisms associated with COPD and pulmonary hypertension. However, due to significant difference in allelic frequencies, we were unable to directly replicate the obtained results.

Another finding of this study indicates that the *TRPA1* rs7819749 polymorphism significantly influences lung function in COPD patients. Correlation analysis showed that the degree of bronchial obstruction had a tendency to aggravation with the increase in the number of the T alleles. In addition, we replicated the effect of *TRPM8* rs11562975 on lung function that was previously found in smoking asthma patients [33]. There are no previous studies that estimated the influence of *TRPM8* and *TRPA1* polymorphisms on lung function in COPD, so the identified associations are now reported for the first time. Gallo et al. [31] and Kotova et al. [43] described linkage between *TRPA1* polymorphisms and the development of asthma in children and adults. However, in the former study the T allele of rs959974 increased the risk of childhood asthma, while we found that it was rather protective against excessive deterioration of lung function in COPD. *TRPM8* rs11562975 is another polymorphism with well documented functional significance. The C allele was consistently reported to determine increased sensitivity to local skin cooling [44,45], bronchoconstriction in response to cold air inhalation [27] or severe bronchial obstruction in smoking asthma patients [33]. These results are in perfect agreement with the data obtained in this study—the C allele carriers were prone to enhanced bronchial obstruction as measured by FEV1/FVC ratio and significantly prevailed among COPD patients with post-bronchodilator FEV1 < 60% independently on the other factors.

Finally, we unexpectedly found that *TRPA1* rs959976 and *TRPM8* rs17865682 polymorphisms may alter the airway response to β2-adrenergic agonist salbutamol. It is difficult to draw firm conclusions regarding the molecular mechanisms explaining this effect, although interactions between G protein-coupled receptors and TRP channels are quite common [46]. It was also established that rs959976 is associated with migraine onset before the age of 15 and can modulate the response of *TRPA1* to coal fly ash [32,47].

Relatively small sample size is the main limitation of this study. Nevertheless, substantiated hypothesis and choice of polymorphisms, consistent significance of the obtained associations after correction for possible confounding factors and repetitive confirmation of *TRPM8* rs11562975 effect in independent samples increase the likelihood of the results’ reliability.

## 5. Conclusions

In conclusion, in the present study we discovered the variants of *TRPM8* and *TRPA1* genes that may probably influence COPD predisposition and lung function in European population which suggests the potential importance of these cationic channels in COPD pathogenesis. The heterozygous genotype of *TRPM8* rs2052030 tended to protect smokers from COPD development. In COPD patients, *TRPA1* rs7819749 influenced the degree of bronchial obstruction measured both before and after the inhalation of bronchodilator. In addition, we replicated the effect of *TRPM8* rs11562975 on lung function and established the associations of *TRPA1* rs959976 and *TRPM8* rs17865682 polymorphisms with changes in salbutamol response. Future studies are suggested not only to verify the role of *TRPM8* and *TRPA1* polymorphisms in a larger sample but also to provide functional evidence for the observed effect of allelic variants at molecular level.

## Figures and Tables

**Table 1:** Brief characteristics of the studied polymorphisms.

**RefSNP No.**	**Gene**	**Chromosome** **and Position (GRCh38)**	**Region**	**Nucleotide (Amino-acid) Substitution**	**Selection** **Criteria**
rs7577262	*TRPM8*	2:233910224	5′-near gene	g.233910224G >A	[22,23]
rs10166942	*TRPM8*	2:233916448	5′-near gene	c.-990T > C	[24,25,26]
rs11562975	*TRPM8*	2:233945906	exon	c.750G > C (p.Leu250 =)	[27,28]
rs2052030	*TRPM8*	2:234018966	3′-UTR	c.*1710C > G	possible miRNA binding site
rs17865682	*TRPM8*	2:234019581	3′-near gene	c.*2325A > G	[29]
rs920829	*TRPA1*	8:72065468	exon	c.535G > A (p.Glu179Lys)	[30,31]
rs7819749	*TRPA1*	8:72063566	exon	c.558A > C (p.Lys186Asn)	missense SNP, possible exonic splicing silencer
rs4738202	*TRPA1*	8:72028626	intron	c.2937+1275T > C	[31]
rs959976	*TRPA1*	8:72023910	exon	c.3053A > G (p.His1018Arg)	[32]
rs959974	*TRPA1*	8:72023604	intron	c.3149+210C > A	[31]
rs6996723	*TRPA1*	8:72021397	3′-UTR	c.*1509G > A	possible miRNA binding site

**Table 2:** Clinical and Functional Characteristics of the Studied Groups.

Characteristics	Chronic Obstructive Pulmonary Disease (COPD) (*n* = 143)	Healthy Smokers (*n* = 104)	*p*-Value
Age (years)	61.0 (56.0–66.0)	43.5 (34.5–52.0)	<0.001
Gender (M/F, %)	85/15	74/26	0.03
Pack-year index	40.0 (26.0–45.0)	15.0 (6.0–22.5)	<0.001
Pack-year ≥ 10 (%)	97	67	<0.001
Forced expiratory volume in one second (FEV1) (% pred.)	40.2 (30.3–58.1)	100.0 (92.8–114.5)	<0.001
Forced vital capacity (FVC) (% pred.)	60.1 (47.0–75.7)	104.5 (96.0–116.5)	<0.001
FEV1/FVC (%)	46.2 (38.0–54.2)	80.4 (76.1–85.2)	<0.001
Peak expiratory flow (PEF) (% pred.)	41.0 (31.7–55.0)	95.2 (86.5–111.0)	<0.001
Forced expiratory flow (FEF)50 (% pred.)	13.0 (8.0–23.5)	87.5 (73.0–101.5)	<0.001
FEF75 (% pred.)	11.9 (1.4–20.9)	76.7 (60.7–88.8)	<0.001
Maximum mid-expiratory flow (MMEF) (% pred.)	15.0 (11.1–23.5)	85.8 (68.7–100.0)	<0.001
∆FEV1 (%)	13.7 (5.0–23.0)	5.1 (2.5–8.8) ^1^	<0.001
∆FEV1/FVC (%)	4.2 (0.1–9.9)	2.3 (0–5.0) ^1^	0.05
PostBD-FEV1 (% pred.)	48.0 (36.4–62.5)	103.1 (94.3–115.0) ^1^	<0.001
PostBD-FEV1/FVC (%)	48.3 (40.4–55.7)	79.8 (75.0–82.7) ^1^	<0.001

Data are presented as median (interquartile range) or as percentage. ^1^ Response to bronchodilator was assessed in 42 control subjects only.

**Table 3:** Genotype frequencies of the studied polymorphisms in COPD patients and control group.

Polymorphism	Genotypes	COPD	Healthy Smokers	*p*-Value	*p*-Value Adjusted ^2^	False Discovery Rate (FDR) *p*
*TRPM8* rs7577262 ^1^	GG	118 (82.5%)	82 (78.8%)	0.47	0.79	
	GA	24 (16.8%)	19 (18.3%)			0.98
	AA	1 (0.7%)	3 (2.9%)			
*TRPM8* rs10166942 ^1^	TT	96 (67.1%)	69 (66.3%)	0.89	0.79	
	TC	40 (28.0%)	31 (29.8%)			0.98
	CC	7 (4.9%)	4 (3.9%)			
*TRPM8* rs11562975 ^1^	GG	113 (79.0%)	86 (82.7%)	0.47	0.49	
	GC	28 (19.6%)	17 (16.3%)			0.92
	CC	2 (1.4%)	1 (1.0%)			
*TRPM8* rs2052030	CC	81 (56.6%)	44 (42.3%)	0.01	0.08	
	CG	48 (33.6%)	54 (51.9%)			0.30
	GG	14 (9.8%)	6 (5.8%)			
*TRPM8* rs17865682 ^1^	AA	105 (73.4%)	75 (72.1%)	0.82	0.61	
	AG	33 (23.1%)	27 (26.0%)			0.98
	GG	5 (3.5%)	2 (1.9%)			
*TRPA1* rs920829 ^1^	AA	2 (1.4%)	3 (2.9%)	0.10	0.15	
	AG	30 (21.0%)	30 (28.8%)			0.43
	GG	111 (77.6%)	71 (68.3%)			
*TRPA1* rs7819749	GG	60 (42.0%)	46 (44.2%)	0.87	0.98	
	GT	68 (47.5%)	49 (47.1%)			0.98
	TT	15 (10.5%)	9 (8.7%)			
*TRPA1* rs4738202	AA	9 (6.3%)	8 (7.7%)	0.71	0.85	
	AG	51 (35.7%)	41 (39.4%)			0.98
	GG	83 (58.0%)	55 (52.9%)			
*TRPA1* rs959976 ^1^	AA	92 (64.3%)	61 (58.7%)	0.36	0.17	
	AG	44 (30.8%)	39 (37.5%)			0.43
	GG	7 (4.9%)	4 (3.8%)			
*TRPA1* rs959974	GG	28 (19.6%)	20 (19.2%)	0.86	0.97	
	GT	71 (49.6%)	55 (52.9%)			0.98
	TT	44 (30.8%)	29 (27.9%)			
*TRPA1* rs6996723 ^1^	GG	107 (74.8%)	77 (74.0%)	0.89	0.43	
	GA	34 (23.8%)	25 (24.0%)			0.92
	AA	2 (1.4%)	2 (2.0%)			

^1^ Polymorphisms with less than five observations for minor allele homozygotes in either group were analyzed in the dominant model; ^2^ after adjustment for sex, age and pack-year index.

**Table 4:** Association Between *TRPM8* rs2052030 and COPD Under Different Genetic Models.

Genetic Model	Genotypes	OR (95%CI)	*p*-Value	OR (95%CI) Adjusted ^1^	*p*-Value Adjusted ^1^	FDR *p*
Dominant	CC vs. CG+GG	0.56 (0.34–0.94)	0.03	0.47 (0.24–0.93)	0.03	0.22
Recessive	CC+CG vs. GG	1.77 (0.66–4.78)	0.34	0.94 (0.23–3.80)	0.93	0.98
Over-dominant	CC+GG vs. CG	0.47 (0.28–0.79)	0.004	0.47 (0.24–0.93)	0.03	0.22
Log-additive		0.78 (0.52–1.15)	0.21	0.60 (0.35–1.04)	0.07	0.30

^1^ After adjustment for sex, age and pack-year index.

## Data Availability

The data supporting this study are available on request from the corresponding author.

## Supplementary Materials

The following are available online at https://www.mdpi.com/2075-4426/11/2/108/s1, Table S1: Detailed conditions of *TRPM8* and *TRPA1* polymorphisms genotyping.
