# Supplementary material for: Effect of TRPM8 and TRPA1 Polymorphisms on COPD Predisposition and Lung Function in COPD Patients
Source: J Pers Med. 2021 Feb 8;11(2):108. doi: 10.3390/jpm11020108 (PMC7915134; doi:10.3390/jpm11020108)
Supplement: Supplementary file 1 [file jpm-11-00108-s001.pdf]

Table S1. Detailed conditions of *TRPM8* and *TRPA1* polymorphisms genotyping.

Polymorphism	Primers and Probes	C, μM	Ta_1, °C	Ta_2, °C
TRPM8 rs7577262	FWD 5′-CCTCAAAAGCCCAGTTCCCCTAACCTCA-3′	0.02	65	62
	REV 5′-GTAAAGAATCAGTAAATGTGAACCACT-3′	0.5		
	Probe 5′-FAM-CTCCGAATGCAGTTTCCTCTCGGAG-BHQ1-3′	0.5		
TRPM8 rs10166942	FWD 5′-TACTTACTACCTAACACTTGGC-3′	0.5	64	58
	REV 5′-TGAGCAAGGTCTGAAAGGAAGGATAGGG-3′	0.02		
	Probe 5′-FAM-CGCGTAACAAAGAGAGACAAAAGCGCG-BHQ1-3′	0.5		
TRPM8 rs11562975	FWD 5′-CCAGTACCTTATGGATG-ACTT-3′	0.5	62	58
	REV 5′-GGAGCTTTGCTTCGACAGTGGGAT-3′	0.02		
	Probe 5′-FAM-CGGCCAGGATATACAGTGGAGCCG-BHQ1-3′	0.5		
TRPM8 rs2052030	FWD 5′-GGAGAGATTATCTTACTGAACAC-3′	0.5	62	58
	REV 5′-TCACTACTGCCCAGACAAAAGGAAAA-3′	0.02		
	Probe 5′-FAM-CGGCTCAAATACGACCACTGCCG-BHQ1-3	0.5		
TRPM8 rs17865682	FWD 5′-GAATTGTCATGTGTTGCTTTTG-3′	0.5	62	60
	REV 5′-AGAGAAGTCAGTTCATGTAACTCTGGGA-3′	0.02		
	Probe 5′-FAM-CCGGAAGTGAATCTGACCGG-BHQ1-3′	0.5		
TRPA1 rs920829	FWD 5′-TGAATAAAAGATAAACAGACATGGTCCCT-3′	0.02	60	58
	REV 5′-AGAAAGCAGACAGATATGACA-3′	0.5		
	Probe 5′-FAM-CTGGCACCACAAATAATA-GCGAGCCAG-BHQ1-3′	0.5		
TRPA1 rs7819749	FWD 5′-GCGATTTGGGGTTTAACTGGTGTCAT-3′	0.02	63	61
	REV 5′-GCAGCTTGGTGAATAGGGA-3′	0.5		
	Probe 5′-FAM-CTTGCTTAACAAAGGAGCAAG-BHQ1-3′	0.5		
TRPA1 rs4738202	FWD 5′-GCCTACTTAGCACACCAG-3′	0.5	63	60
	REV 5′-TTCCAATCGCTCTGTGTCTCTGTATAATA-3′	0.02		
	Probe 5′-FAM-CCGGTCATAGAACATAGAATCCGG-BHQ1-3′	0.5		
TRPA1 rs959976	FWD 5′-TAGCAGATTGTGGGTTCCTTGAGGGCATA-3′	0.02	66	60
	REV 5′-AGATTTATCAGCATTTGGTATTTCTTG-3′	0.5		
	Probe 5′-FAM-CGCGTTTTTATCCGACAGCATACGCG-BHQ1-3′	0.5		
TRPA1 rs959974	FWD 5′-AACATATTTGCCACTATTCACCAATCAGTT-3′	0.02	63	59
	REV 5′-TTACTCCAGGGATCTTCAG-3′	0.5		
	Probe 5′-FAM-CCGGGCCTTCCAATCATAACCGG-BHQ1-3′	0.5		
TRPA1 rs6996723	FWD 5′-AATCACTATACAGAAGCAATATCC-3′	0.5	62	57
	REV 5′-TTCTTGGCACTCAGTGAATGTTGACT-3′	0.02		
	Probe 5′-FAM-CCTCCCCCTTGTCTTTATGGAGG-BHQ1-3′	0.5		
